# Australian settlement workers’ use of food security information resources with refugee clients: a qualitative exploration

**DOI:** 10.1017/S1368980025101766

**Published:** 2026-01-02

**Authors:** Julie Maree Wood, Alison Booth, Rebecca Lindberg, Claire Margerison

**Affiliations:** Institute for Physical Activity and Nutrition, School of Exercise and Nutrition Sciences, Deakin University, Geelong, Australia

**Keywords:** Refugees, Food insecurity, Nutrition, Food culture, Information resources

## Abstract

**Objective:**

Refugees are susceptible to food insecurity. In high-income countries (HIC), settlement workers (SW) provide information, including food security information resources, to newly arrived refugees. Australia has a range of resources, but their use in settlement work is unknown. This study’s aims were to explore SW’s resource use with refugee clients.

**Design:**

This descriptive, qualitative study explored SW’s perceptions regarding resource use. One-on-one interviews, using a semi-structured guide, were conducted. The Technology Acceptance Model’s usage constructs (including Actual Use and Perceived Usefulness) informed the guide and analytical constructs. Under these constructs, emergent usage themes were identified.

**Setting:**

Six Australian cities.

**Participants:**

Settlement workers.

**Results:**

Fourteen workers were interviewed. Thirteen worked for government-related departments. Most used resources as part of client welcome packs to address acute food insecurity and/or support clinical deficiency issues. Print, pictorial, translated and co-designed resources were perceived to be most useful. Less useful were resources with limited cultural tailoring, translation issues and high literacy demand. There was limited use of digital resources due to variations in clients’ digital access and literacy. Opportunities for improvement include streamlining access, addressing topics such as clinical deficiencies related to food insecurity and increasing culturally nuanced translation.

**Conclusions:**

Development of culturally appropriate resources, facilitating resource access and improved food culture information may help SW better support refugee populations with food security challenges during resettlement in HIC.

The number of refugees around the world has increased year-on-year for the last 12 years^(1)^. In 2023, there was an 8 % increase, equating to 8·8 million people^(1)^. In mid-2024, high-income countries (HIC) hosted nearly 30 % of the world’s refugees^(2)^. During resettlement in HIC, people living as refugees bring with them many strengths and skills but, in the unfamiliar environment, they also face many challenges^(3)^. One challenge is food insecurity^(4)^. Food insecurity exists when there are limitations in food availability, access, utilisation, agency, stability and sustainability^(5)^. During resettlement, people living as refugees are highly susceptible to food insecurity^(4,6,7)^. A 2023 Australian study reported that the prevalence of food insecurity amongst refugee populations was nearly double that of the general population^(4)^.

The consequences of food insecurity include poorer health, academic, developmental, behavioural, socio-familial and mental health outcomes and an increased risk of nutrition-related conditions, such as anaemia^(8,9)^. Determinants that threaten food security for migrant populations include temporal economic issues, language and cultural barriers, limited transport access and a new food environment where food acquisition, preparation and food types are often unfamiliar or culturally unacceptable^(10,11)^. For refugees, these determinants can be further exacerbated by literacy issues, poor access to buffering factors such as social networks and pecuniary disadvantage^(10,11)^.

Many food insecurity determinants, such as economic disadvantage, may be outside of an individual’s control during early resettlement, but other factors, such as transport use, language barriers and navigation of the new food environment, may be addressed when there is access to suitable information and support. Refugee access to quality settlement services and information regarding all aspects of the new environment is vital during early resettlement^(12,13)^. A 2018 study reported that possession of information was a primary enabler of refugee integration^(14)^. A 2020 US study exploring refugee and immigrant health information needs (*n* 85) reported that nutrition information was at the top of participants’ self-reported responses, with 88 % selecting it^(15)^.

Settlement workers (SW) are the frontline of settlement service provision and information access for refugees in HIC, such as Australia. In Australia’s settlement environment, the term ‘refugee’ is used to describe a person who holds an Australian humanitarian visa and is in their early settlement period, often deemed to be the first 5 years following arrival^(16)^. This paper will use the term ‘refugee’ in the same manner. The SW delivering Australia’s early settlement service provide refugees with essentials, such as healthcare, and facilitate access to information regarding the new environment^(17)^. A 2016 study reported that SW were a key information source for people who are forcibly displaced^(18)^. This includes food and nutrition information, particularly for SW directly involved in healthcare and health promotion^(15)^.

A 2025 content analysis established that the Australian food security-related resource collection (*n* 184) developed for migrant and refugee populations is mostly current, accurate and suitable, but their *in situ* use and efficacy have not been explored^(19)^. With the increasing need for HIC to resettle refugees, and an established need for quality information and services during resettlement, it is important to now understand the use of these resources in the settlement environment^(12,18,20)^. This knowledge may then be used to understand the resource needs of SW in their work with refugee clients and also to inform studies exploring the use and perceived effectiveness of the resources by refugee populations.

The aim of this study is therefore to explore SW’s perceptions regarding the use of the current food security-related information resources and to understand SW’s perceptions of gaps and opportunities for developing information resources to better assist refugees with food security issues.

## Methodology and methods

### Study design

This exploratory, qualitative study aimed to investigate the perceptions of SW, that is, their feelings, thoughts and experiences, regarding resource use. For each SW, their reality may influence their attitude towards, and use of, resources in subjective ways. The methodological approach had to reflect this. Therefore, an interpretivist epistemological approach was appropriate as interpretivism assumes that facts are entwined with values and experiences and, therefore, there are multiple realities^(21)^. This aligns with a relativist ontological position and subjective realism perspective. This perspective determines that there is not one ‘correct’ reality; rather, reality is perceived by the participant^(22)^. This ontological position is appropriate for studies which explore participants’ experiences as it focuses on the perceptions and experiences of participants, not the positivist pursuit of an absolute truth^(23)^.

This manuscript reports on one facet of a larger exploratory qualitative study that investigated (i) food security challenges that refugee clients present to service providers and (ii) SW use of information resources with their clients. The results related to food security challenges presented are reported in a separate paper^(24)^. In this manuscript, the analysis focuses on the use of food security-related information resources (herewith resources). This includes any resource which addresses one or more of the FAO’s food security dimensions (agency, availability, access, utilisation, sustainability and stability), clinical nutrition deficiencies related to food insecurity and/or components of food literacy^(25)^. Examples include resources focused on topics such as acute food relief/food assistance, cultural food access, navigation of Australia’s food environments, identification and use of local fresh produce, transport information, navigation and use of kitchen appliances in Australia, and food budgeting and planning in Australia.

This study was conducted according to the guidelines laid down in the Declaration of Helsinki, and all procedures involving research study participants were approved by the Deakin University Human Ethics Advisory Group (HEAG-H 43_2024). Written informed consent was obtained from all subjects. The study’s method and results adhered to the consolidated criteria for reporting qualitative research (COREQ) checklist^(26)^.

### Researcher positionality

The research team is comprised of Australian women from Anglo-Celtic backgrounds, all of whom have tertiary qualifications in dietetics and/or nutrition. The study included cross-cultural elements, and so there was a risk of cross-cultural misrecognition^(27)^. To mitigate the risk of this, the study design included culturally reflexive practices such as cross-cultural training and, for the interviewer, the maintenance of a reflexive journal^(28)^.

### Recruitment

A national database of 140 refugee settlement organisations was developed using publicly available contact details. The list included all of Australia’s government settlement-related agencies and non-government organisations (NGO) that deliver primary care and settlement services to refugees^(17)^. The database was used to recruit potential participants. A combination of convenience and snowball sampling was used^(23)^. An invitation to participate was emailed out to all service providers on the database. If no response was received, a reminder was sent 2 weeks after the initial email. Interested individuals were emailed a Plain Language Statement and consent form prior to the commencement of their interview. Signed consent forms were received by the research team prior to interviews. At the commencement of interviews, receipt of consent forms was confirmed, and verbal consent was also recorded. To compensate them for their time, participants were offered a $50 grocery voucher.

### Participants

To be eligible, SW had to be working directly with refugees during their early settlement period. Eligible participant roles included medical professionals, community nutritionists, dietitians, community development or health promotion officers, and settlement officers.

### Data collection

Data were gathered via one-on-one interviews with participants. Interviews were conducted in English, online, via Zoom, by the lead researcher (JMW). Interviews were planned to take between 45 and 60 min. The interviewer maintained field notes. At the interview’s conclusion, participants were invited to provide feedback or review the transcript^(23)^. The interview period ran from June to August 2024. With participant consent, all interviews were audio-recorded, and an initial transcript was automatically generated. At the conclusion of each interview, the recording was used to verify and make minor edits to ensure transcript accuracy. Audio files were stored on a password-protected data storage repository.

### Study tools and measures

As the aim was to explore SW perceptions using an interpretivist and subjective realism approach, the primary study tool was a semi-structured interview guide. The semi-structured nature of the guide allowed the researcher to build rapport with the SW and explore their reality. The interview guide covered five domains: demographics, client description, food security issues presenting, resource use, and future gaps and opportunities (Table 1).

**Table 1:** Semi-structured interview guide

Domain	Indicative questions
Demographics and role	Participant background and experience
Description of clients/patients	Country of birth, settlement timeframe.
Food security issues^*^	Food security issues presenting to settlement service providers
Resource use and usefulness (including actual use, perceived usefulness, attitude towards using, external variables)	Do you find the resources available are useful for the sorts of food security issues your clients/patients talk to you about? (enablers, barriers, external variables) How could information resources be more useful to you? (enablers, barriers, external variables, intention to use)
Resource ease of use (including perceived ease of use, external variables)	Are the resources easy to use? Are there things that make it hard to use them? (barriers, enablers, external variables)
Gaps and opportunities (including behavioural intention to use)	How do you think information resources could be *more useful* to you in with your work with food insecure refugee clients? What could be done to make the resources *easier for you to use with your clients*?

* Food security issues are reported in a separate paper^(24)^.

Constructs from the Technology Acceptance Model (TAM) were used to inform the study’s semi-structured interview guide and, during analysis, as usage category headings under which emergent themes were presented. TAM is comprised of six usage headings or constructs: actual use; behavioural intention to use; attitude towards using; perceived usefulness; perceived ease of use; and external variables. The model has theoretical foundations in social psychology’s theory of planned behaviour and theory of reasonable action^(29)^. It was used in this study as it is a widely accepted usage model in healthcare research, has been used to test use of online as well as print resources and provided usage constructs or headings but allowed the participant’s realities to emerge under each of the usage constructs^(30,31)^.

### Data analysis

#### Statistical analysis

Participants’ demographic characteristics were noted in Excel and then imported into IBM SPSS (version 29). The data were reported using medians, means, standard deviations, frequencies and percentages.

#### Thematic analysis

Braun and Clarke’s process for analysing qualitative data was used to guide the analysis process, along with other established tools^(32–34)^. Throughout the interview period, transcripts and field notes were read, re-read and discussed by the research team^(33)^. This in-field analysis was designed to familiarise the team with the data and track code and meaning saturation as the interview period progressed^(34)^. Meaning and code saturation were discussed regularly throughout the interview period. After twelve interviews, new data began to support a consensus on views and key words were repeated. The subsequent final two interviews did not result in new codes.

De-identified transcripts were imported into NVivo 15 (QSR International, 2024).

TAM constructs were utilised as ‘usage’ headings. The headings used were actual use, attitude towards using, perceived usefulness, perceived ease of use, external variables, and gaps and opportunities (incorporating behavioural intention to use). The data were analysed deductively, and themes were developed under the TAM headings. To minimise bias, two coders independently coded 25 % of the transcripts (*n* 4). Intercoder agreement was then assessed by discussing the coded data from the randomly chosen interviews. There was good intercoder agreement on code content, but there were some minor discrepancies related to theme labels. Label naming was agreed by consensus, and coding was then completed. Throughout the coding process, the research team discussed the themes under each TAM usage construct, and exemplar quotes were selected^(32)^. The audit trail, NVivo files and coding process were then reviewed by RL before coding was finalised.

## Results

### Participant characteristics

All national settlement providers on the database (*n* 140) were emailed invitations to participate in the study. This included ninety-five government and forty-five NGO settlement providers. Of those initially contacted, six SW agreed to be interviewed, thirty-seven forwarded the email or referred other relevant settlement organisations, nine declined to participate as they did not work directly with refugees and eighty-eight did not respond (including fifty government agencies and thirty-eight NGO). A further eight participants were recruited via the referrals. Fourteen interviews were conducted. Interviews ranged from 35 to 65 min, with a mean duration of 48 min (±9·4 sd). Nearly, all participants worked for government service agencies or providers. Nearly, a third of participants had lived refugee experience (*n* 4), most were female (79 %, *n* 11), and all were health professionals. Experience working with refugee populations ranged from 6 months to 20 years, with a median of 6 years. Participants worked with populations originating from numerous countries, but Myanmar and Afghanistan were the most frequently reported (see Table 2).

**Table 2:** Characteristics of refugee settlement service worker participants (*n* 14)

	*n*	%
Gender		
Female	11	79
Male	3	21
Settlement provider type		
Government agency or department	13	93
Non-governmental organisation	1	7
State^*^		
Western Australia	5	36
New South Wales	3	21
Queensland	3	21
Tasmania	2	14
Victoria	1	7
Work role and experience		
Role^†^		
Medical professional specialising in refugee health	6	43
Dietitians	3	21
Health education officers	3	21
Public health nutritionists	2	14
Setting^‡^		
Community	4	29
Clinic	4	29
Clinic and in-home^†^	3	21
Clinic and community	2	14
In-home	1	7
Years of experience working with people living as refugees		
Less than 1 year	2	14
1–4 years	3	21
5–9 years	5	36
10–14 years	1	7
15+ years	3	21
Refugee clients/patients they work with		
Country of birth^§^		
Afghanistan	12	86
Myanmar	11	79
Syria	6	43
Democratic Republic of Congo	6	43
Ethiopia	6	43
Eritrea	5	36
Iran	3	21
Iraq	2	14
Honduras	2	14
Ecuador	2	14
Venezuela	2	14
Rwanda	1	7
Ukraine	1	7
Bhutan	1	7
Somalia	1	7
Settlement timeframe in which they provide services		
First 6 months after arrival	1	7
0–1 year of settlement	3	21
0–2 years of settlement	5	36
0–5 years of settlement	3	21
0–7 years of settlement	1	7
Up to 10 years of settlement	1	7

* In Australia, states are similar to counties. The setting in which they work with people living as refugees.

† Medical professionals included doctors and nurses working directly with refugees during early resettlement.

‡ The services or consultations take place in the home of the person.

§ Percentages total more than 100 as participants worked with multiple populations in terms of country of birth.

### Themes related to perceived resource use and usefulness

The thematic analysis generated themes under each TAM construct. These themes are presented below.

#### Technology Acceptance Model construct: Actual Use

##### Actual use theme: widespread usage but purpose and delivery method varied

All of the interviewed SW reported using food and nutrition information resources with their refugee clients, but the way in which they were used varied. Many used them to support clients presenting with specific clinical nutrition concerns (such as vitamin B_12_ or calcium deficiencies) related to food security issues. Most also used resources with clients who reported specific acute food security issues, although for clients with low literacy, navigation of food relief directories was perceived to be challenging. Some SW used resources as a routine part of their clients’ arrival packs to build local food literacy and food system navigation awareness. This was whether the client reported food security issues or not.

*‘Those Brimbank resources from Victoria were great at showing how to buy fruit and veges at the supermarket and how to cook different [local] vegetables. Those sorts of things are really useful’*. (P03, Public Health Nutritionist)

The way in which resources were delivered or introduced *in situ* also varied amongst SW. Some SW reported that they included them in the welcome packs or general information folders but did not refer to them during the appointments. Other SW reported that they preferred to introduce the resources during appointments, particularly if an interpreter was present. The facilitated introduction increased the SW perceived resource usefulness, particularly for clients with low literacy, *‘we’ll give the English, and then our interpreters will go through it with them in [their] language’* (P02, Medical Professional). Another settlement worker felt a facilitated introduction assisted with behaviour change,

*‘Demonstrate what it is that the information is providing and… show them at the same time, they’re more likely to remember or to adopt that behaviour’*. (P13, Public Health Nutritionist)

#### Technology Acceptance Model construct: Attitude Towards Using

Increased scope and capacity was the central theme related to Attitude Towards Using.

##### Attitude towards using theme 1: increased scope and capacity

For most, their attitude towards using resources was linked to the resources’ perceived ability to build SW’s practice scope and capacity beyond allocated appointment time. Many of the SW felt that their time with refugee clients was limited, and there were numerous acute settlement issues to address within the limited time frame. Some also perceived that their clients were stressed by pre-arrival experiences, local settlement issues and/or ongoing issues with family overseas. The SW reported that the physical print resources provided them with the opportunity to give their patients important food and nutrition information that could be accessed and utilised by all members of the household when time allowed, when they were less stressed or when necessity dictated.

*‘We can’t verbalise every single bit of information to people, especially because in that first few weeks of arriving they’ve got so much to think about… health is often down at the bottom… so I hand them a bunch of resources’*. (P08, Medical Professional)

#### Technology Acceptance Model construct: Perceived Usefulness

All SW reported that resources were useful when working with refugee clients. Two themes facilitated this perceived usefulness: simple; user-friendly format and co-design. One theme challenged it: lack of alignment with clients’ needs and capabilities.

##### Perceived usefulness theme 1: print, pictorial, translated formats facilitate usefulness

Print, pictorial and translated resources were widely perceived to be the most useful. All SW perceived print resources to be *‘really helpful’* (P08, Medical Professional). It was the preferred format as print was perceived to maintain an ongoing presence in the home and was accessible to other household members,

*‘I might only see the mum when I’m doing the visit… if it’s sitting on the kitchen table…other family members might have a little look’*. (P04, Medical Professional)

One settlement worker noted that in larger households, *‘sometimes the shopping gets done by not necessarily the mum’* (P04, Medical Professional), so at home the physical print resource can reach the household grocery buyer.

Many SW’s perception was that usefulness was improved when they could *‘provide resources in their languages, in Arabic, in Burmese…’* (P09, Health Education Officer). This was not always possible as SW reported that there was a perceived lag between the development of translated resources and the languages of current incoming refugee waves. However, SW also reported that literacy was very low amongst some cultures, and so text-based resources, translated or not, were not useful for all cultures, *‘lots of our Burmese clients don’t speak or read Burmese’* (P06, Dietitian). For this reason, there was a preference for pictorial food and nutrition resources, as they *‘can speak to anyone’* (P04, Medical Professional). One settlement worker noted that being pictorial-based, *‘makes them transferable…it’s not uncommon for me to have clients who can’t write or aren’t comfortable reading and it just makes them easy and accessible’* (P06, Dietitian). Another settlement worker noted that there were 135 language groups in Myanmar, some with very low literacy, but amongst these, some food cultures were similar and so pictorial resources may be used across some these linguistically diverse groups. Another settlement worker provided an example of how pictures can be used to convey dietary-related meaning across similar food cultures,

*‘…in Asian cuisine, for example, there’s those small rice bowls, so they use them as a measuring device to show portion sizes… that’s a good example of using very limited language to show a dietary pattern’.* (P06, Dietitian)

##### Perceived usefulness theme 2: co-design facilitates usefulness

Co-design of resources also improved their perceived usefulness as there was community ownership, and they were culturally appropriate and relevant,

*‘We start developing our resources from bottom up, because …we want the community to use the resources. So, our community leaders spend a lot of time looking at what the traditional fruits are, what their ingredients are, and then substituting some of the ingredient that can be substituted with a cheaper, healthy options’.* (P11, Health Education Officer)

Another settlemement worker noted that if you don’t co-design, *‘you don’t understand their reality in their context…you don’t see from their lens’* (P11, Health Education Officer). Using co-design, their team had developed a catalogue of resources that the community welcomed,

*‘…. because of the collectivist nature of the community, it was working really well… you need to get 5, 6 people to do something, and then everyone else is doing it…so it’s going viral’.* (P11, Health Education)

##### Perceived usefulness theme 2: lack of alignment with client needs and capabilities limits usefulness

Many SW perceived that a lack of alignment with their clients’ needs and capabilities impeded resource usefulness. This included a perceived lack of cultural tailoring, translation issues, limited topic range and misalignment with client literacy capabilities. The overarching cultural tailoring and translation perception was that, often, translated resources were just *‘English words turned into a different language’* (P05, Dietitian) and were text-heavy and culturally inappropriate, *‘just Western advice, translated’* (P05, Dietitian). Explaining why culture had to be an intrinsic part of resource translation, one SW said that in some community groups, *‘there is no word for vegetable… they just eat, they eat food’* (P13, Public Health Nutritionist). Also related to culture and translation, many SW perceived the use of abstract concepts or jargonistic terms, such as ‘serves’, to compromise the usefulness of the resources,

*‘How many serves you have? If you look at one of the cultural foods … I’m from there, [the] Ethiopian/Eritrean plate. It is a plate, you share food, and there are a lot of different foods in there… So how do you know how many serves you’re getting in that plate? So that is a biggest challenge for our families’.* (P03, Medical Professional)

Limited topic range was also perceived to curtail resource usefulness. Topic gaps ranged from *‘emergency food relief directories in language’* (P05, Dietitian) to nutritional deficiencies resulting from chronic food insecurity, with one settlement worker saying,

*‘A lot of the time we’re doing like high energy, high protein diets, trying to get kids that are malnourished to gain weight… we’re after very pictorial resources, unless they’re translated and even if they translated, I find them very heavy in detail’.* (P10, Dietitian)

The high literacy demand of many resources was also perceived to limit usefulness for some cultural groups, *‘40 % of our families are illiterate in their first language in certain ethnic groups’* (P02, Medical Professional). Most SW spoke about this disparity in literacy levels across different cultural groups,

*‘In terms of food security, it’s really difficult because you’ve got all these wonderful directories and pages, leading you to food emergency relief, but if you can’t read it, and you can’t talk over the phone in English, then they’re very limited in their use, especially with those who are very food insecure’.* (P05, Dietitian)

Conversely, in other cultures, whose pathway to settlement included access to education, one settlement worker noted, *‘you have literature professors, you have people who are tertiary educated, worked as specialists in their home country’* (P02, Medical Professional), so amongst these groups, English and/or translated resources were perceived as highly useful.

#### Technology Acceptance Model construct: Perceived Ease of Use

Under Perceived Ease of Use, recurring use of the same resource cache was perceived as a facilitator and difficulty locating new resources was a challenge.

##### Perceived ease of use theme 1: recurring use of the same resource cache facilitates ease

Most SW had a curated cache of resources they repeatedly used, *‘I’ve got all the links saved’* (P08, Medical Professional) and *‘I tend to use the same ones all the time’.* (P05, Dietitian). Therefore, most reported resources to be easy to use.

##### Perceived ease of use theme 2: difficulty finding new resources challenged ease

Beyond these curated caches, many reported that finding new resources takes too much time, *‘we actually spend a lot of hours trying to find where they are’* (P11, Health Education Officer) and because of this, others in the healthcare team rely on them to find resources,

*‘If everything could just be in one place, one easy to use website’.* (P13, Medical Professional)

When SW could not find what they needed, many opted to make custom resources,

*‘Our refugee health nurses, they do a bit of lunchbox talk as well, but we’ve resourced them with that, so we realized that’s a gap, and that’s a food security issue’.* (P13, Medical Professional)

#### Technology Acceptance Model construct: External Variables

Three themes were related to External Variables: easy access, dissemination barriers and digital barriers.

##### External variable theme 1: easy access facilitates use

Some SW reported having access to an intranet of resources that they could readily access and use with their clients. This facilitated both use and ease of use.

##### External variable theme 2: dissemination barriers limit use

Many SW reported that they were unable to disseminate resources beyond their organisation due to lengthy approvals processes,

*‘The barriers are… working for the government and being able to publish something, takes a really long time’.* (P13, Public Health Nutritionist)

##### External variables theme 3: digital barriers limit use

Digital barriers limited use due to costs associated with digital access, low trust in digital platforms and variable digital literacy levels across different cultural groups.

Most SW rarely used digital resources due to their clients’ hesitancy to use digital media because of associated costs, *‘for most of our families having wi-fi is an issue’* (P02, Medical Professional). Low trust in digital platforms was also a factor which limited use with concern amongst clients regarding scams, so they would not open email attachments.

**Figure 1:**
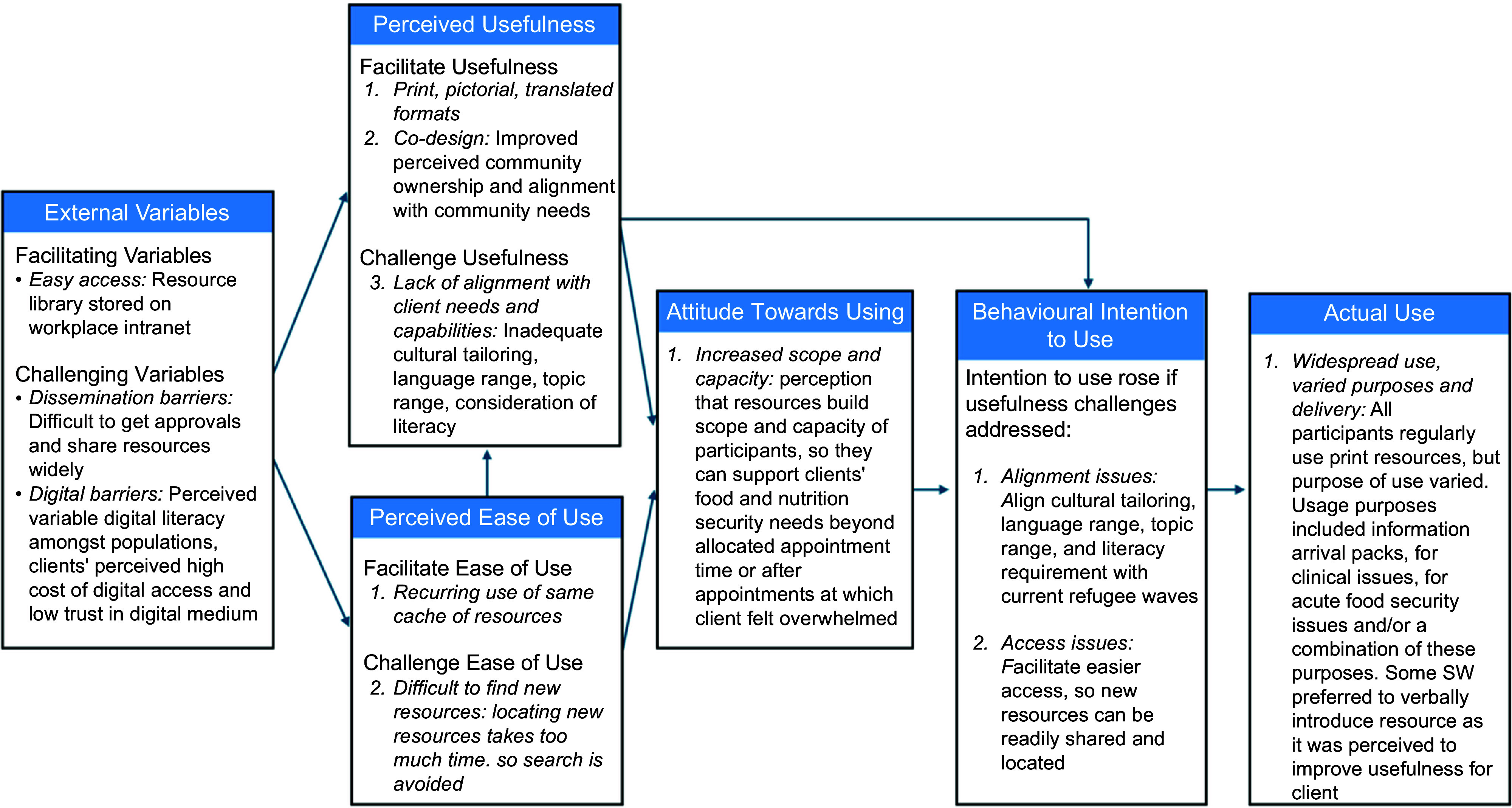
Settlement workers’ perceived use of food security-related information resources, reported using the Technology Acceptance Model constructs. Reference: Davis FD. Perceived usefulness, perceived ease of use and user acceptance of information technology. Manage Inf Syst Q 1989; 13: 319–340. https://pubmed.ncbi.nlm.nih.gov/?term=13*[volume]+AND++319[page]+AND++1989[pdat].

Few SW reported routinely using digital resources as they did not have the physical in-home presence of print, and many agreed that ‘*digital literacy is an issue’* (P01, Health Education Officer) in many populations. Even when working with digitally literate clients, most SW preferred to use print as well, *‘I would have to go into their phone, set up the pinpoint. They’d have to remember to go back to it. So, if I give them a handout, at least they know what it looks like on the map’* (P06, Dietitian). If an SW showed clients a digital resource, such as a video, many would augment it with print as they *‘would like to leave them with something’* (P07, Medical Professional) or *‘if it’s just been shared on a phone to one person, it might not be shared amongst the whole family as much’* (P04, Medical Professional). Figure 1 shows results related to TAM constructs.

### Gaps and opportunities

Most SW agreed that *‘there’s a lot of gaps’* (P05, Dietitian), and there was an intention to use resources more if gaps were addressed. There were six key areas where SW thought resources could be improved: access, media type, topic range, translation, cultural and SW support (see online Supplemental Material).

Most SW wanted easier access to resources and for existing websites to more effectively categorise resources, so they could easily find what they need. Many wanted a central repository dedicated to refugee food and nutrition resources,

*‘If there was one central place for nutrition resources…I mean you could be broad enough to be culturally, linguistically diverse, but I think just for refugee communities’* (P13, Public Health Nutritionist).

There was consensus amongst most SW that print was still required, but digital should be considered for acute food access, including translated cultural food directories, and for populations with low fundamental literacy. The use of digital resources, such as videos, was being introduced in one SW’s health team for use on practitioner iPads and to be played in healthcare waiting rooms, which may be *‘useful for the [accompanying] interpreter too*’ (P04, Medical Professional).

SW outlined specific topics they would like added to the resource range, particularly for emerging refugee populations. These included resources to address clinical issues, specifically malnutrition, iron, calcium and vitamin deficiencies, resulting from pre-arrival food insecurity. Other topics included food budgeting, tap water safety and information regarding dairy’s dietary role and how to buy it. Translated resources to build food literacy were also discussed, but this was dependent on the prevailing level of fundamental and food literacy amongst each culture.

SW wanted the translation to include cultural nuance (including food culture) and consideration of literacy levels, English text to be included on translated versions so they are easier to update, and more extensive use of simple text, action words and pictures. Minimising conceptual complexity was also recommended. Rather than resources featuring ‘serves’, SW wanted to know how to apply the Australian Dietary Guidelines and Guide to Healthy Eating to, for example, a *‘Syrian one-pot family stew’* or an Eritrean share plate.

SW also thought use could be improved if resources were easier to share with the national SW network, particularly the primary healthcare and nutrition network. One settlement worker’s refugee health team had an extensive custom range of culturally and linguistically translated, co-designed resources but were not sure how to share them, *‘they are within our health service, and I’m not sure if you can access them on our intranet’* (P02, Medical Professional). There was also a call for health authorities to align resource development with current incoming refugee waves, in terms of culture, language, literacy and food and nutrition information needs. This included providing SW with food culture information regarding the current waves,

*‘In this refugee space, there’s not a lot of dietitians, and there’s not a lot of crossover… I feel like it’s very limited and the national refugee nutrition network is not just dietitians and not clinical…we probably need a more clinical-led stream as well, so that you could talk to more people that way’.* (P10, Dietitian)

Finally, there was a call for more support for SW to develop their own resources when required, *‘if you’re putting together resources for people from a non-English speaking background…things to consider…a bit of a checklist’.* (P03, Public Health Nutritionist)

## Discussion

This study aimed to explore SW perceptions regarding the usefulness of the food security-related information resources and to identify opportunities to improve resources. Under the TAM Actual Use construct, the SW reported that they routinely used print resources, but most used the same cache of resources on a recurring basis. While usage was widespread, usage behaviour did vary amongst SW in the way they delivered resources to their clients and the purpose for which they used the resources. Under TAM’s Perceived Usefulness construct, print, pictorial, and translated formats, and co-design were important components of usefulness. The central theme which challenged usefulness was a lack of perceived alignment with clients’ needs and capabilities. Recurring use of the same resource cache was the primary theme under the TAM Perceived Ease of Use construct, and difficulty locating new resources was the primary challenge. Under the TAM External Variables construct, easy access facilitated use and dissemination, and digital barriers challenged it. Opportunities to improve resource usefulness and increase intention to use included making access easier, continuing print but considering digital, addressing specific topic gaps, more culturally nuanced translation and more resources to support SW.

SW perceived resources to be more useful when they were in print and were co-designed with the refugee community. These are findings well supported by the literature^(35,36)^. A 2021 scoping review of nutrition education strategies for refugees in HIC reported that a collaborative approach improved programme design^(35)^. A 2022 study evaluating nutrition digital resources amongst newly arrived migrants (*n* 85) found a significant improvement in healthy eating knowledge amongst three of the four language groups but no change in the Karen-speaking participants^(37)^. Post-test focus group discussions revealed that the Karen group contained Karen, Karenni and Burmese people, all with different languages and dialects. As a result, the video was not understood by most. Co-design may not have guaranteed improved results, but it may have assisted with cultural and linguistic nuance and lived experience insight during resource development and, thereby, influenced the usefulness of the resources^(35)^.

Under Actual Use, SW preference for print resources was, in part, due to the enduring physical presence of the resource in the home, exposing it to the wider household. This is a component of distributive health literacy^(38)^. A 2024 study of SW and Myanmar refugees’ health literacy and implications for nutrition and dietetic practice noted that distributive or community literacy played a central role in decision-making in the collective culture^(39)^. A 2022 study of Bhutanese refugees reported similar findings^(38)^. The print resources’ role in distributive health literacy requires more understanding. However, these findings do suggest that the physical presence of the print resource positions it as a tool that may leverage distributive health literacy and, therefore, help to ensure accurate information is disseminated throughout the community.

Results suggest that, for some populations, a culturally nuanced translation of the English may be appropriate, but, for others, an entirely different approach (message and media) may be required. The literature reports a similar finding. Nur et al.’s 2021 review found that literacy level, culture and language proficiency are common barriers to nutrition education^(35)^. The study also reported the importance of developing context-specific models to ensure relevance to specific cultures. For example, many food literacy capabilities are contextual^(40)^, so while food literacy may be low in the new food environment, people living as refugees may arrive with high levels of food literacy in their native or transition food environment^(41)^. Further research to understand the transferability of food literacy on arrival could provide the foundations for strengths-based food literacy programmes for refugees during early resettlement.

The SW interviews revealed a trove of lived experience, cultural insight and the existence of large libraries of co-designed, culturally nuanced resources locked within silos in different states’ (similar to counties) systems. Some SW had lived experience as refugees and deep understanding of the food cultures and dietary patterns that other SW were seeking more support to understand. A US study of settlement caseworkers also called for more support, specifically to improve SW’s capacity to help clients adapt to the new food environment and address food security challenges^(42)^. Similarly, a 2016 report called for more support and resources for clinicians working with refugees to help them adapt practice to differing levels of patient health literacy^(43)^. The development of SW-targeted information resources, such as cultural profiles and dietary patterns, may provide more support for SW when seeking to mitigate refugee clients’ food security challenges.

While resources are only one part of the multidisciplinary, multifaceted approach required to address food insecurity amongst refugee populations, SW perceive them to be a valuable component of their work with food-insecure refugee clients. It is therefore important that the library of resources is readily accessible, relevant and reflective of the current needs of SW and the refugee populations they support. Further understanding of the food cultures across Australia’s different refugee populations would also be valuable to assist SW with understanding dietary patterns and food culture nuances to inform clinical practice and food security support. With this insight into SW use of resources, it is next essential to understand the perceived use and efficacy of these resources among refugee populations. Finally, the co-design of resources to fill the gaps outlined by SW and refugees would ensure the bank of resources available are fit for purpose.

### Strengths and limitations

To the authors’ knowledge, there are no studies focused on SW use of nutrition resources with multiple refugee populations. This study therefore helps to build knowledge in this area as, during early resettlement, SW are known to be a primary source of trusted information^(1)^. Further, this study provides insight into how SW engage and use the nutrition information environment for their refugee clients and patients. It identifies ways in which food and nutrition resources can be improved and SW can be better supported to assist food-insecure refugee clients.

A limitation of this study is that nearly all of the SW interviewed worked for government organisations, yet settlement services are also delivered by NGO in Australia’ settlement network. The study did include SW from six of Australia’s seven states, so it does provide some national insight, but, with only one NGO participant, the findings may not be as applicable to the NGO in the settlement space. Further to this, the qualitative study design and use of a convenience sample may limit the generalisability of the results. Rather, the results may be indicative and may therefore be used to inform future studies and resource development strategies and health/refugee communication research and services more broadly. A further limitation is the risk of social desirability bias influencing actual use results. To mitigate this, participants were interviewed individually and context regarding use was presented in study results to provide insight into usage behaviours rather than just the widespread frequency of usage finding.

### Conclusion

This study’s SW reported widespread use of print food security-related information resources with their refugee clients. Most SW use a recurring cache of resources with a strong preference for print, pictorial and translated versions. Locating new and relevant resources was a key challenge to increasing usage. The SW identified numerous gaps and opportunities to improve the access to the range in terms of literacy requirement, topic range and location of the resources. It is now important to build on this knowledge and understand the perspective of refugee populations in terms of how they use these resources and the role they may play in addressing their food security issues *in situ*. These resources are an existing asset and, in a changeable funding environment, it would be beneficial to understand how best to utilise this asset to better meet the food security needs of these priority populations.

## Supporting information

Wood et al. supplementary material Wood et al. supplementary material

## Supplementary material

For supplementary material accompanying this paper visit https://doi.org/10.1017/S1368980025101766
