# Supplementary material for: Australian settlement workers’ use of food security information resources with refugee clients: a qualitative exploration
Source: Public Health Nutr. 2026 Jan 2;29(1):e14. doi: 10.1017/S1368980025101766 (PMC12895447; doi:10.1017/S1368980025101766)
Supplement: Wood et al. supplementary material [file S1368980025101766sup001.docx]

**Supplementary Material: Gaps and Opportunities Identified for Improved Use of Resources**


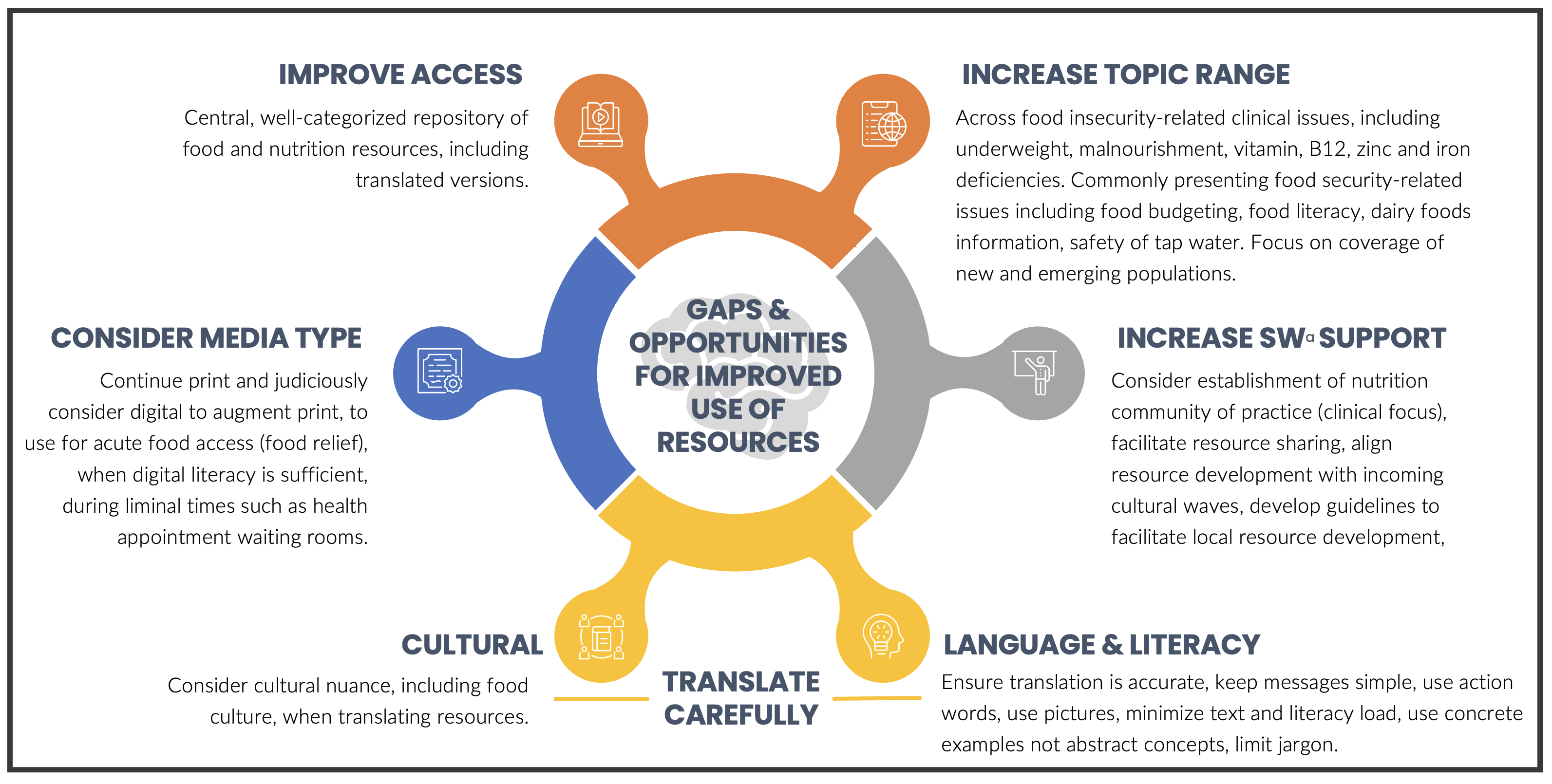


^a^ SW= settlement worker
